# Identification of AC025811.3 and AC012354.6 as two critical survival-related lncRNAs for uterine corpus cancer

**DOI:** 10.1371/journal.pone.0351323

**Published:** 2026-06-12

**Authors:** Lu Pu, Rui Ou, Zhaomin Deng, Hao Jiang

**Affiliations:** 1 Laboratory for Aging and Cancer Research, National Clinical Research Center for Geriatrics, West China Hospital, Sichuan University, Chengdu, Sichuan, China; 2 Laboratory for Aging and Cancer Research, Frontiers Science Center for Disease-related Molecular Network, West China Hospital, Sichuan University, Chengdu, China; 3 School of Sports Medicine and Health, Chengdu Sport University, Chengdu, China; 4 Department of Laboratory Medicine, Clinical Laboratory Medicine Research Center, West China Hospital, Sichuan University, Sichuan Clinical Research Center for Laboratory Medicine, Chengdu, China; 5 Department of Gastroenterology and Hepatology, West China Hospital, Sichuan University, Chengdu, China; 6 Sichuan University-University of Oxford Huaxi Joint Centre for Gastrointestinal Cancer, Frontiers Science Center for Disease-Related Molecular Network, West China Hospital, Sichuan University, Chengdu, China; CSIR-Indian Institute of Chemical Biology, INDIA

## Abstract

Uterine corpus endometrial carcinoma (UCEC) ranks as the most frequently diagnosed gynecologic malignancy and the second leading cause of gynecologic cancer-related mortality. Long non-coding RNAs (lncRNAs) have emerged as critical regulators of gene expression and tumor biology; however, their prognostic significance in UCEC remains largely unexplored. To systematically identify survival-associated lncRNA biomarkers, we integrated clinical and transcriptomic data from two independent cohorts: TCGA-UCEC (548 tumor, 35 normal) and CPTAC-Uterus (102 tumor, 15 normal). Following upper quartile normalization and ComBat-based batch effect correction, differentially expressed lncRNAs (FDR < 0.01, |log2FC| > 2) were identified using Student’s t-test. The intersecting set of consistently dysregulated lncRNAs from both cohorts was subjected to Cox proportional hazards regression to identify survival-associated candidates. Functional inference was performed through Spearman correlation with protein-coding genes and Gene Set Enrichment Analysis (GSEA) of KEGG pathways. A total of 550 lncRNAs were consistently downregulated and 148 were upregulated in UCEC across both cohorts. Cox regression identified 30 survival-associated lncRNAs (FDR < 0.01), all with elevated expression correlating with worse overall survival. The top two candidates, AC025811.3 and AC012354.6, showed significant stage-dependent expression patterns across FIGO stages I–IV and were functionally enriched in immune regulation and carbohydrate metabolism pathways. In conclusions, AC025811.3 and AC012354.6 represent novel candidate prognostic lncRNA biomarkers in UCEC. Experimental validation, including FISH-based tissue localization and staged quantification, and functional assays, is warranted to confirm their biological roles.

## Introduction

Uterine corpus endometrial carcinoma (UCEC) is the most frequently diagnosed gynecologic malignancy and ranks second in gynecologic cancer mortality. In the United States, an estimated 67,880 new cases were diagnosed and 13,250 deaths were attributed to this disease in 2024 [1–4]. Notably, incidence and mortality rates have been steadily increasing. In contrast to improvements in survival for many other common cancers since the mid-1970s, survival outcomes for uterine corpus cancer have deteriorated. From 2007 to 2016, the mortality rate increased by 1.9% per year on average [5–7]. Significant disparities in 5-year relative survival are also evident across racial groups, with Black individuals experiencing poorer outcomes. These trends may be partially attributed to factors such as rising obesity rates, aging populations, and reduced use of combined menopausal hormone therapy [8–10]. However, the specific genomic alterations driving these adverse trends remain largely undefined, highlighting a critical gap in our understanding of uterine corpus cancer progression.

However, the specific genomic alterations driving these adverse trends remain largely undefined, highlighting a critical gap in our understanding of uterine corpus cancer progression [11]. Long non-coding RNAs (lncRNAs), defined as non-coding transcripts exceeding 200 nucleotides in length, were historically considered transcriptional “noise”. However, recent research has revealed their significant regulatory potential, particularly in cancer [12]. leading to increased interest in lncRNAs as therapeutic targets. Aberrant expression, mutations, and altered methylation status of lncRNAs have been implicated in various cancer types. Indeed, several lncRNAs have been shown to play key roles in gynecologic malignancies, including breast and cervical cancers. For example, HOTAIR is upregulated in breast cancer, promoting invasiveness and metastasis by targeting chromatin repressor Polycomb proteins [13]. LINK-A mediates HIF1α stabilization, HIF1α-p300 interaction, and activation of HIF1α transcriptional programs, which promotes breast cancer glycolysis reprogramming and tumorigenesis [14]. Similarly, CCHE1 is significantly upregulated in cervical cancer tissues, and its depletion inhibits cervical cancer cell proliferation [15]. Further investigation revealed that CCHE1 physically associates with proliferating cell nuclear antigen messenger RNA, thereby enhancing its expression. Despite these advances, limited research has focused on tumor-related lncRNAs in UCEC. Thus, while lncRNAs are increasingly recognized as critical regulators in tumor initiation and progression, their specific mechanisms and functions in UCEC remain largely unexplored.

In this study, we aimed to identify survival-associated lncRNAs in uterine corpus cancer using publicly available genomic data. The recent release of Clinical Proteomic Tumor Analysis Consortium (CPTAC) genomic data through the NCI’s Genomic Data Commons (GDC), including 322 cases from bronchus/lung, kidney, and uterus, provides a valuable resource for this research. We specifically utilized 101 cases of uterine cancer within the CPTAC dataset. To minimize false positive findings, we also incorporated TCGA-UCEC (Uterine Corpus Endometrial Carcinoma) datasets, consisting of 560 uterine cancer cases. Using these combined datasets, we identified lncRNAs with altered expression in uterine cancer and subsequently prioritized those associated with patient survival. Finally, we predicted the potential biological functions of these survival-related lncRNAs.

## Results

### Batch effect correction and data normalization

**Fig 1:**
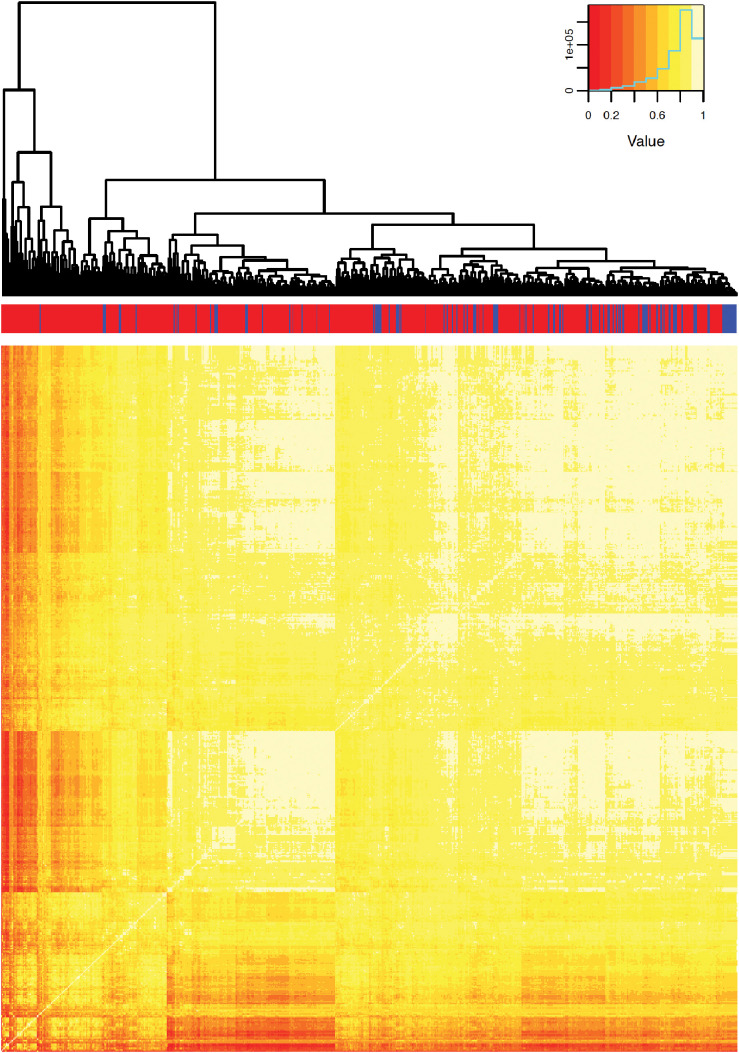
Spearman correlation heatmap of samples (A) before and (B) after batch effect correction. The color scale represents the Spearman correlation coefficient, as indicated by the color bar. Row side colors indicate the sample source: red for TCGA-UCEC and blue for CPTAC-Uterus.

To address experimental variations arising from differences in time, laboratory, and protocols (batch effects), we applied upper quartile normalization to the TCGA-UCEC and CPTAC-Uterus datasets. Spearman correlation analysis, visualized through heatmaps (Fig 1A), revealed distinct clustering of samples by dataset origin (TCGA vs. CPTAC), regardless of sample type, indicative of significant batch effects. To mitigate these effects and improve data comparability, we implemented ComBat, a batch effect correction algorithm [16]. Following ComBat correction, Spearman correlations between TCGA-UCEC and CPTAC-Uterus samples were reduced (Fig 1B), demonstrating effective batch effect removal.

### Expression profiles of protein-coding genes and lncRNAs

**Fig 2:**
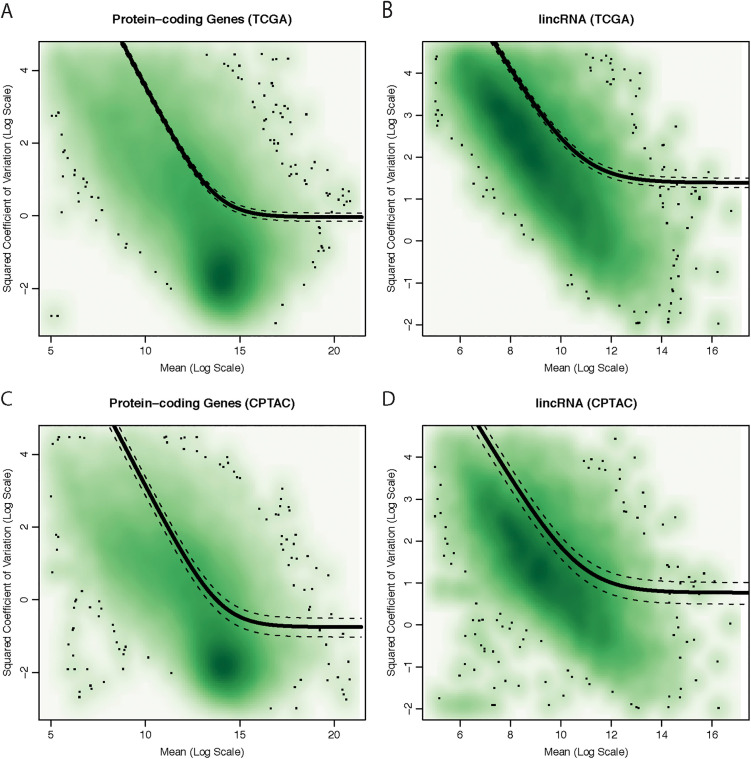
Expression profiles of protein-coding genes and lncRNAs in TCGA-UCEC and CPTAC-Uterus datasets, illustrating the relationship between mean expression and variability. The plots display the squared coefficient of variation (CV²) versus the mean expression (both on a log scale) for protein-coding genes and lncRNAs in TCGA-UCEC (A and B) and CPTAC-Uterus (C and D). These plots highlight differences in the distribution of expression levels and variability between the two gene classes (protein-coding and lncRNA) and across the two datasets.

We then analyzed the overall expression profiles of protein-coding genes and lncRNAs within the normalized datasets (Fig 2). Protein-coding genes exhibited similar expression profiles in both TCGA-UCEC and CPTAC-Uterus, with a mean expression level of approximately 14 and a low squared coefficient of variation (approximately −2) (Fig 2A and 2C). These findings suggest that the majority of protein-coding genes are expressed at similar levels across samples, with minimal inter-sample variation. In contrast, lncRNA expression profiles differed significantly from those of protein-coding genes, although TCGA-UCEC and CPTAC-Uterus shared similar patterns. LncRNAs generally exhibited lower expression levels compared to protein-coding genes and displayed substantially greater inter-sample variation. This heightened variability in lncRNA expression may contribute to observed differences in patient survival outcomes.

### Differentially expressed lncRNAs in UCEC

**Fig 3:**
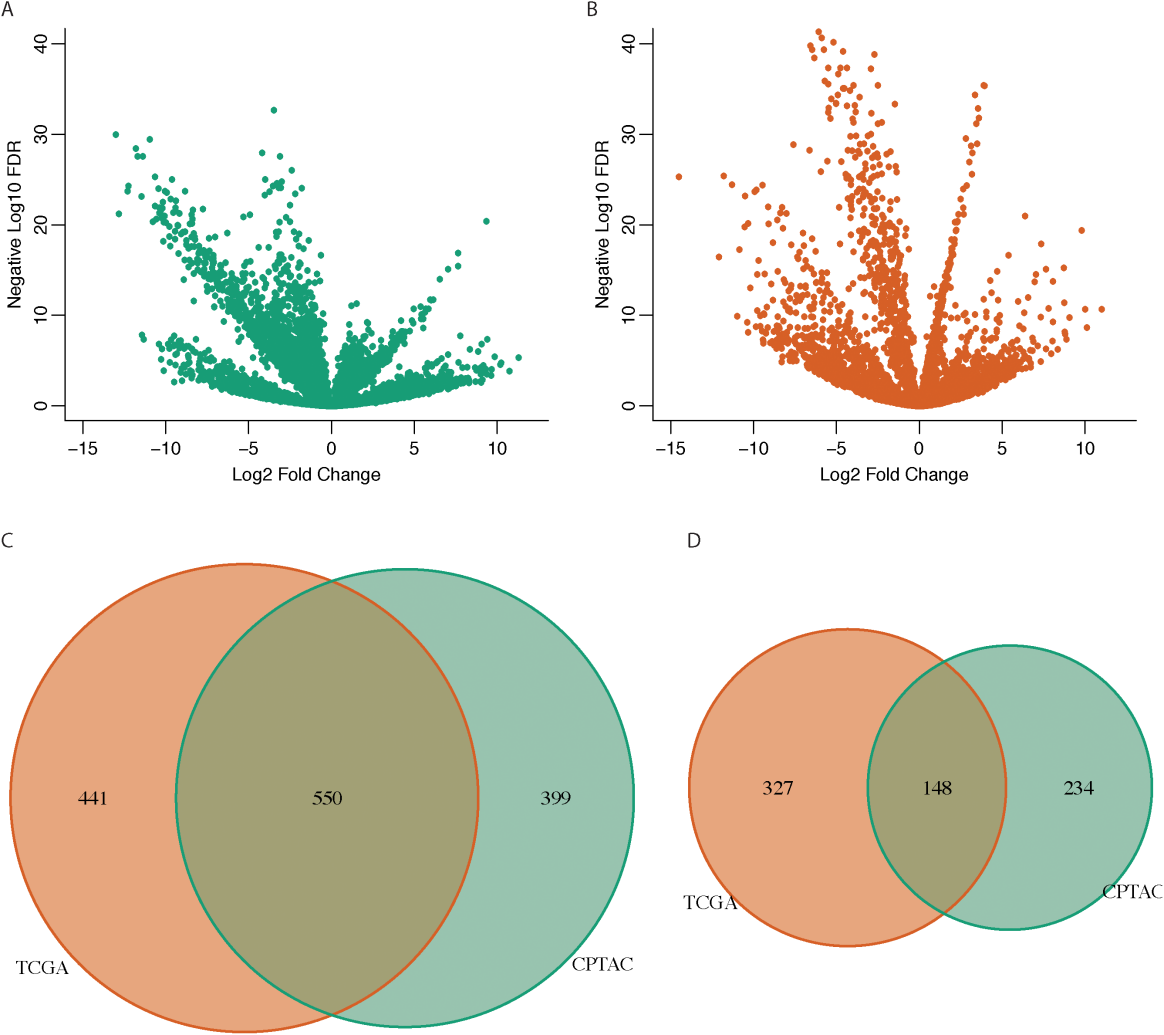
Identification of differentially expressed lncRNAs between tumor and normal samples in TCGA-UCEC and CPTAC-Uterus datasets. Volcano plots display the relationship between log2 fold change and negative log10 FDR for lncRNA expression in TCGA-UCEC (A) and CPTAC-Uterus (B). Venn diagrams illustrate the overlap of downregulated (C) and upregulated (D) lncRNAs identified in the TCGA-UCEC (green) and CPTAC-Uterus (orange) datasets.

To identify lncRNAs differentially expressed between tumor and normal samples, we performed Student’s t-tests on the TCGA-UCEC and CPTAC-Uterus datasets. TCGA-UCEC comprised 548 tumor samples and 35 normal samples, while CPTAC-Uterus included 102 tumor samples and 15 normal samples. We defined differentially expressed lncRNAs as those with a false discovery rate (FDR) < 0.01 and an absolute log2 fold change > 2. In TCGA-UCEC, we identified 991 downregulated and 475 upregulated lncRNAs in tumor samples (Fig 3A and 3B).

We then examined the overlap between differentially expressed lncRNAs identified in TCGA-UCEC and CPTAC-Uterus. The overlap of downregulated lncRNAs was less than 60% between the two datasets (Fig 3C), and the overlap of upregulated lncRNAs was less than 40% (Fig 3D). Although these overlaps were statistically significant according to bootstrapping analysis, the relatively low concordance suggests a potentially high false positive rate for identifying tumor-related lncRNAs. This discrepancy may be attributable to the limited number of normal tissue samples, biases in sample populations, or other confounding factors. Therefore, we considered only the intersection of downregulated and upregulated lncRNAs from the two datasets as highly confident differentially expressed lncRNAs for subsequent analyses.

### Survival-related lncRNAs in UCEC

To investigate the association between highly confident tumor-related lncRNAs and patient survival, we performed Cox proportional hazards regression analysis. Given the limited number of uncensored cases in the CPTAC-Uterus dataset (5 out of 101), we focused our survival analysis on the TCGA-UCEC dataset. Cox regression models identified 30 lncRNAs significantly correlated with survival status, with a false discovery rate (FDR) < 0.01. Upregulation of all 30 survival-related lncRNAs was associated with poorer survival outcomes.

**Fig 4:**
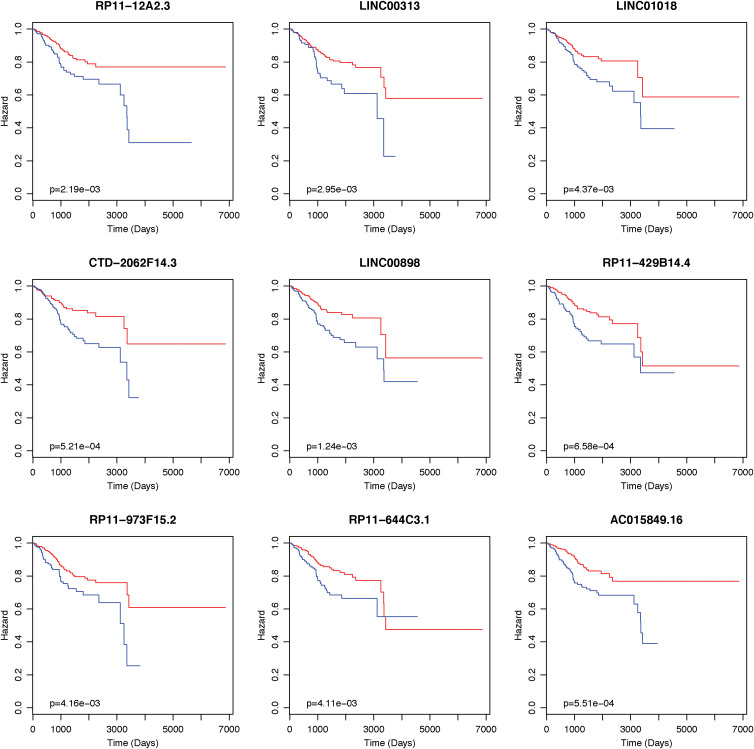
Kaplan-Meier survival curves for selected lncRNAs. Patients were stratified into high-expression (red) and low-expression (blue) groups based on the median expression level of the indicated lncRNA. The p-value for each comparison is indicated in the lower left corner of each plot.

**Fig 5:**
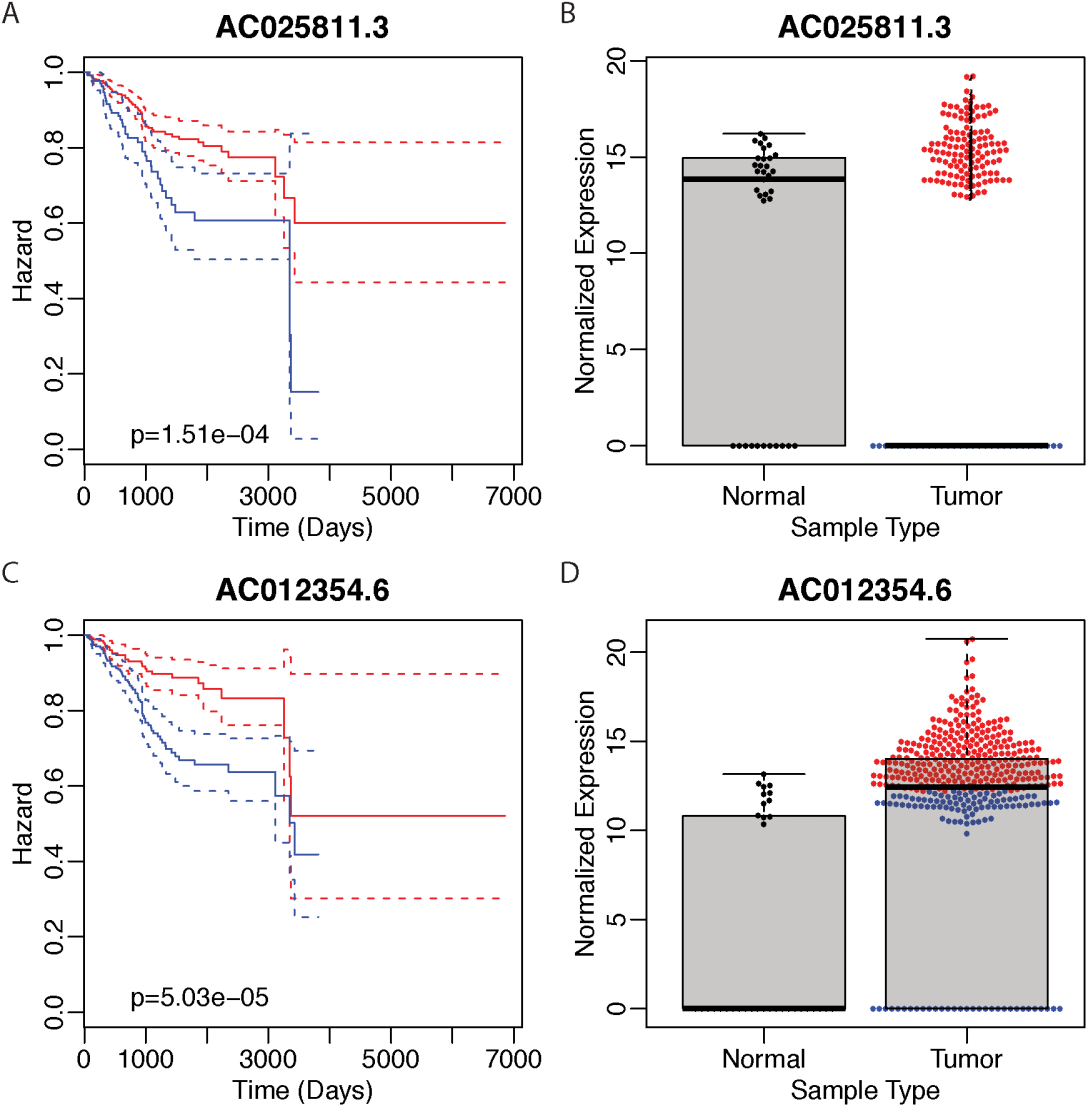
Detailed analysis of two survival-associated lncRNAs, AC025811.3 and AC012354.6. (A and C) Kaplan-Meier survival curves comparing patients with high (red) and low (blue) expression of AC025811.3 and AC012354.6, respectively. Dashed lines represent the 95% confidence intervals. (B and D) Box plots showing the normalized expression levels of AC025811.3 and AC012354.6 in normal and tumor tissues. In the tumor samples, red dots indicate lncRNA expression above the median, while blue dots represent expression below the median. Black dots represent expression levels in normal tissue samples.

To visualize these associations, we stratified samples into two groups based on the median expression level of each lncRNA. Representative Kaplan-Meier survival curves for significant lncRNAs are shown in Figs 4, 5A and 5C.

### Biologically functional analysis for survival-related lncRNAs

To elucidate the potential mechanisms of action for these survival-related lncRNAs, given that the biological functions of most lncRNAs remain largely unknown, we investigated the protein-coding genes correlated with their expression patterns. AC025811.3 and AC012354.6, the two lncRNAs with the lowest false discovery rates (FDRs) in the Cox proportional hazards regression models, were selected as representative models for biological functional analysis.

Kaplan-Meier survival analysis confirmed that median expression levels of both AC025811.3 and AC012354.6 effectively separated patients into distinct survival groups (Fig 5A and 5C). Interestingly, AC025811.3 exhibited low expression in tumor tissues compared to normal tissues; however, higher expression of AC025811.3 within the tumor samples was associated with poorer prognosis (Fig 5B). Conversely, AC012354.6 showed higher expression in tumor tissues, and elevated expression was correlated with decreased survival.

**Fig 6:**
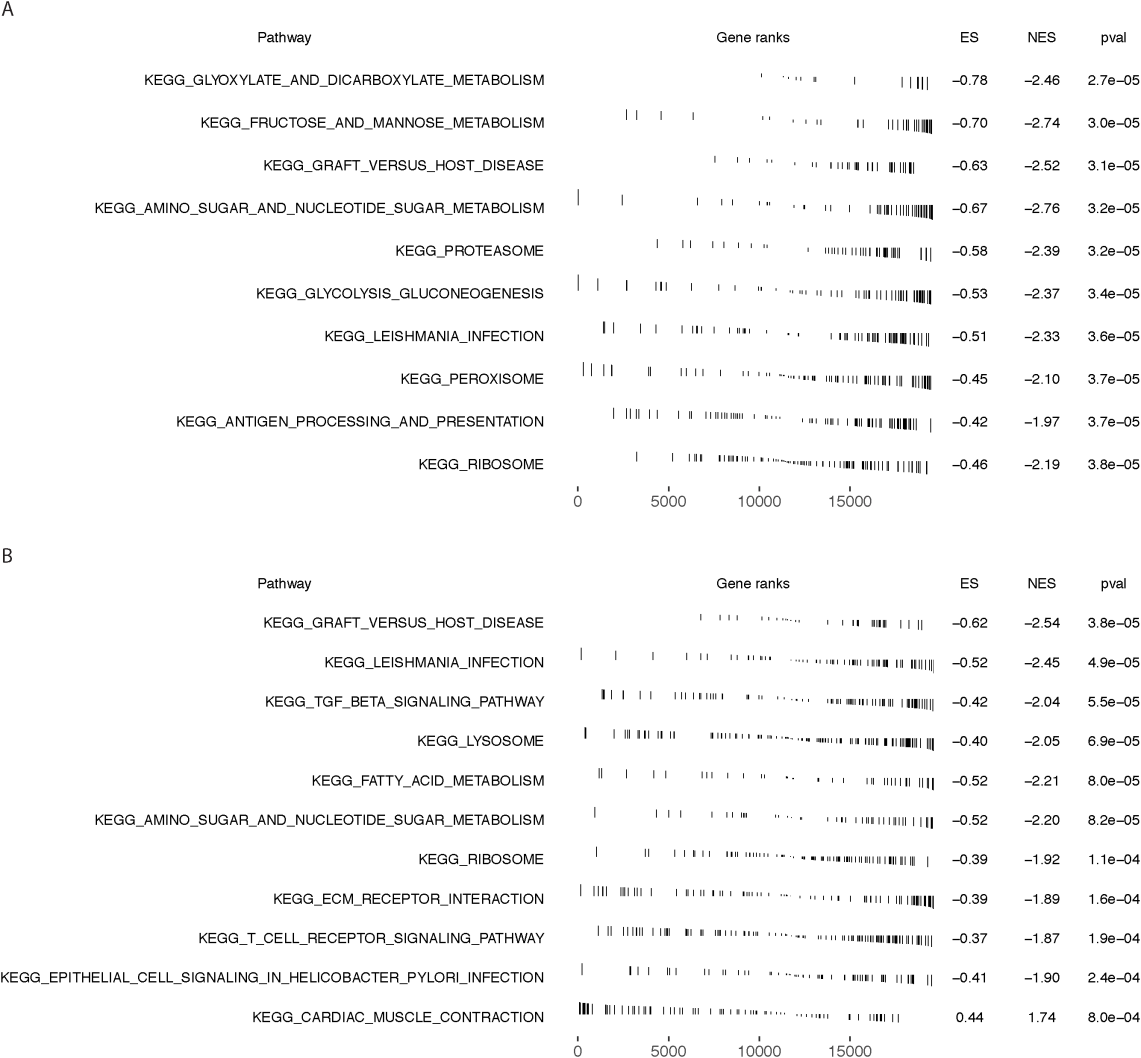
Gene Set Enrichment Analysis (GSEA) plots showing significantly enriched KEGG pathways associated with AC025811.3 (A) and AC012354.6 (B). The plots display the gene rank distribution for each pathway, along with the enrichment score (ES), normalized enrichment score (NES), and p-value.

Since these lncRNAs lack functional annotation, we performed Gene Set Enrichment Analysis (GSEA) to predict their potential roles based on the protein-coding genes correlated with their expression. GSEA revealed shared enrichment of pathways related to immune system processes, including Graft-versus-Host Disease and Leishmania Infection, suggesting a potential role in immune modulation within the tumor microenvironment (Fig 6A and 6B). Additionally, significant enrichment was observed in pathways associated with Ribosome function and Amino Sugar and Nucleotide Sugar Metabolism, indicating a possible involvement in carbohydrate metabolism. While both lncRNAs showed enrichment in these pathways, the presence of distinct, less significantly enriched pathways hints at subtle functional differences. In summary, these findings suggest that AC025811.3 and AC012354.6 function through similar mechanisms, impacting both the immune system and carbohydrate metabolism, with potentially nuanced differences in their specific biological roles in UCEC.

### Stage-stratified expression analysis of AC025811.3 and AC012354.6

**Fig 7:**
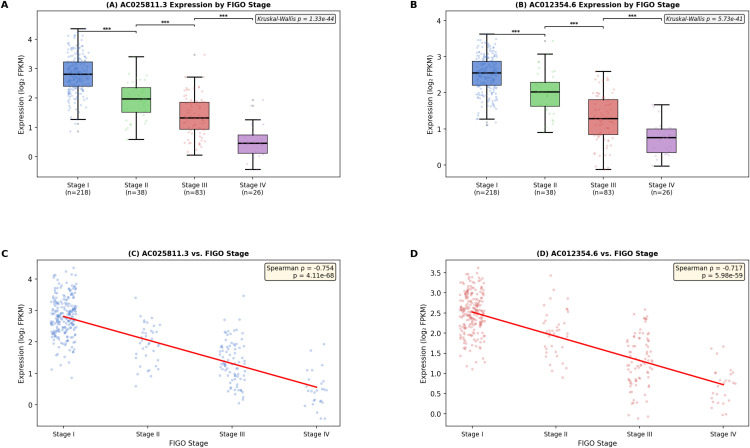
Expression levels of AC025811.3 and AC012354.6 across different pathological stages of uterine corpus endometrial carcinoma (UCEC). (A and B) Box plots showing the normalized expression levels of AC025811.3 (A) and AC012354.6 (B) stratified by FIGO pathological stage (Stage I, II, III, and IV) in the TCGA-UCEC dataset. Expression values are shown as log2-transformed FPKM. Statistical significance between stages was assessed using Kruskal-Wallis test followed by Dunn’s post-hoc test; ns, not significant; *, p < 0.05; **, p < 0.01; ***, p < 0.001. (C and D) Scatter plots illustrating the correlation between AC025811.3 (C) and AC012354.6 (D) expression and FIGO stage as a continuous ordinal variable. Spearman’s rho and p-value are indicated.

Stage-stratified analysis revealed significant associations between AC025811.3 and AC012354.6 expression and FIGO pathological stage (Fig 7). Box plot visualization of TCGA-UCEC data showed differential expression of AC025811.3 across stages I–IV (Kruskal-Wallis test, p < 0.001), with pairwise comparisons indicating significantly lower expression in Stage I compared to Stage III (Dunn’s test, p < 0.01) and Stage IV (p < 0.001). Similarly, AC012354.6 exhibited significant stage-dependent variation (Kruskal-Wallis test, p < 0.001), with higher expression in Stage III compared to Stage I (p < 0.05) and Stage II (p < 0.01). Spearman correlation analysis confirmed positive associations between both lncRNAs and FIGO stage as a continuous variable (AC025811.3: rho = 0.18, p < 0.001; AC012354.6: rho = 0.24, p < 0.001), suggesting that expression levels of both lncRNAs increase with disease progression. These findings support the potential utility of AC025811.3 and AC012354.6 as biomarkers for UCEC staging.

## Discussion

This study set out to identify and characterize novel lncRNA biomarkers associated with patient survival in UCEC, employing an integrative approach that leveraged two independent, publicly available datasets: TCGA-UCEC and CPTAC-Uterus. A key methodological challenge in multi-cohort genomic analyses is the presence of systematic batch effects that may confound biological signal with technical variation. To address this, we applied upper quartile normalization followed by ComBat-based empirical Bayesian batch correction, a well-established approach for harmonizing expression data across platforms [24]. The rationale for requiring lncRNAs to be consistently dysregulated in both datasets was to minimize false positives inherent to single-cohort analyses, given the relatively small number of normal tissue samples in each cohort (35 in TCGA-UCEC; 15 in CPTAC-Uterus). This stringent cross-cohort validation yielded 550 downregulated and 148 upregulated lncRNAs in UCEC, representing a high-confidence set of differentially expressed candidates for further survival analysis.

From this refined set of tumor-related lncRNAs, we identified 30 lncRNAs whose increased expression correlated significantly with poorer overall survival outcomes in UCEC patients. Among these 30 survival-associated lncRNAs, AC025811.3 and AC012354.6 exhibited the lowest false discovery rates (FDRs) in the Cox regression models, suggesting their strong potential as prognostic biomarkers. Due to the lack of direct functional annotation for these lncRNAs, we employed Gene Set Enrichment Analysis (GSEA) of their positively correlated protein-coding gene sets to infer their potential biological roles.

GSEA of protein-coding genes correlated with AC025811.3 and AC012354.6 revealed shared enrichment in immune-related pathways, including Graft-versus-Host Disease and Leishmania Infection, as well as carbohydrate metabolism pathways. The enrichment in immune pathways is consistent with the established notion that the tumor immune microenvironment plays a pivotal role in UCEC progression and patient prognosis. Immune evasion and dysregulated immune cell infiltration have been linked to poor outcomes in endometrial cancer, and lncRNAs have been increasingly recognized as modulators of immune gene expression in various malignancies. The metabolic enrichment is also biologically plausible, as aerobic glycolysis (the Warburg effect) is a hallmark of cancer metabolism, and lncRNAs such as HOTAIR and MALAT1 have been shown to regulate metabolic reprogramming in gynecologic cancers. It is important to note, however, that these GSEA results are inferential and based on co-expression patterns rather than direct functional evidence. The pathway associations therefore represent testable hypotheses for future experimental validation rather than established mechanistic conclusions.

A comprehensive literature review revealed a limited understanding of the functional roles of AC025811.3 and AC012354.6. While no previous studies have specifically investigated AC025811.3, our findings highlight its significant potential as a novel biomarker in UCEC, particularly given its near-complete absence in tumor tissues and its strong association with poor prognosis when expressed. The inverse correlation between AC025811.3 expression and patient survival presents a compelling avenue for future investigation.

Interestingly, previous studies have implicated AC012354.6 in glucose metabolism. A study on glycemic trait loci in China identified a significant association between the rs12712928 variant and the expression level of AC012354.6 [17], suggesting a potential role for this lncRNA in regulating fasting glucose levels. Furthermore, AC012354.6 has been shown to exhibit higher expression levels in mature β-cells compared to in vitro-differentiated insulin-positive β-like cells [18]. Varshney, Scott [19] identified an eQTL variant within AC012354.6 that was significantly associated with both type 2 diabetes and glycemic traits, based on RNA-seq data from pancreatic islets of 112 individuals. These collective findings strongly suggest that AC012354.6 plays a role in glucose metabolism and potentially in the pathogenesis of type 2 diabetes.

Searching lncRNA-related databases, including LncRNADisease [20] and Lnc2Cancer [21], did not yield any direct functional annotations for either AC025811.3 or AC012354.6, highlighting the novelty of our findings and the need for further investigation. However, analysis of miRNA-lncRNA interactions using DIANA-LncBase v2 [22] predicted several potential miRNA binding partners for both lncRNAs, including miR-203b and miR-3618 for AC025811.3 and miR-494 and miR-6738 for AC012354.6. These predicted interactions suggest potential mechanisms by which these lncRNAs could regulate gene expression through competitive binding of miRNAs.

We acknowledge several important limitations of this study. First, our findings are exclusively based on bioinformatics analysis of publicly available TCGA-UCEC and CPTAC-Uterus datasets, and experimental validation has not yet been performed. To substantiate the differential expression claims, fluorescence in situ hybridization (FISH) analysis should be conducted to directly visualize the subcellular localization and expression levels of AC025811.3 and AC012354.6 in paired non-cancerous and cancerous uterine tissue specimens. FISH would provide spatial resolution of lncRNA expression at the single-cell level and help confirm the bioinformatically predicted differential expression patterns in a histological context. Second, the current analysis does not stratify lncRNA expression across the different pathological stages of UCEC (FIGO stages I–IV). A comprehensive stage-wise quantification of AC025811.3 and AC012354.6 expression using quantitative RT-PCR or RNA-seq in a clinically annotated tissue cohort would be essential to determine whether expression levels correlate with disease progression and could serve as a staging biomarker. Third, while the integration of two independent cohorts (TCGA-UCEC and CPTAC-Uterus) improves the robustness of our findings relative to single-dataset analyses, reliance solely on in silico approaches remains insufficient to establish causal relationships. Future experimental studies—including in vitro functional assays (e.g., knockdown and overexpression of AC025811.3 and AC012354.6 in UCEC cell lines) and in vivo xenograft models—are warranted to confirm the biological significance of these lncRNAs. Taken together, the experimental validation outlined above, particularly FISH-based localization studies and stage-stratified expression profiling, will be critical for elevating our current bioinformatics-driven findings to a level of mechanistic and clinical significance.

## Conclusions

In summary, this integrative bioinformatics study identified AC025811.3 and AC012354.6 as candidate survival-associated lncRNAs in UCEC, with elevated expression correlating with poor prognosis in the TCGA-UCEC cohort. Stage-stratified expression analysis further revealed that both lncRNAs show differential expression across FIGO stages I–IV, suggesting potential utility as staging biomarkers. Functional inference through GSEA points to roles in immune modulation and metabolic reprogramming within the tumor microenvironment. We emphasize, however, that these conclusions are based solely on computational analyses of publicly available datasets and should be interpreted with appropriate caution. To translate these bioinformatics findings into clinically actionable knowledge, a multi-tiered experimental validation program is necessary. First, RNA fluorescence in situ hybridization (RNA-FISH) using fluorescently labeled probes against AC025811.3 and AC012354.6 should be performed in formalin-fixed, paraffin-embedded (FFPE) tissue sections from paired non-cancerous endometrium and UCEC tumors. This will confirm differential expression at single-cell resolution and provide spatial context for subcellular localization. Second, quantitative RT-PCR profiling of both lncRNAs across a staged clinical cohort (FIGO stages I–IV) will determine whether their expression correlates with disease progression and whether they have potential as staging biomarkers. Third, functional studies—including siRNA-mediated knockdown and overexpression in UCEC cell lines (e.g., Ishikawa, HEC-1A)—combined with in vivo xenograft models, will be required to establish causal roles in tumor growth, invasion, and immune modulation. Collectively, these steps will bridge the current computational observations with mechanistic and clinical evidence, forming a rigorous basis for evaluating AC025811.3 and AC012354.6 as prognostic biomarkers or therapeutic targets in UCEC.

## Methods

### Description of datasets

Clinical data and gene expression data (mRNA-Seq) from The Cancer Genome Atlas Uterine Corpus Endometrial Carcinoma (TCGA-UCEC) project were downloaded from the GDC Data Portal (retrieved February 16, 2025). In addition, clinical and proteomic data from the Clinical Proteomic Tumor Analysis Consortium (CPTAC)-Uterus project were also obtained; the clinical data were downloaded from the CPTAC Data Coordinating Center Portal because a comprehensive clinical file was not available on the GDC. The CPTAC data used in this publication were generated by the Clinical Proteomic Tumor Analysis Consortium (NCI/NIH). For mRNA-Seq data from TCGA-UCEC, Upper Quartile normalized FPKM (UQ-FPKM) values, aligned to the GRCh38/hg38 human reference genome, were used. The TCGA-UCEC dataset comprised 548 tumor and 35 normal samples from 555 cases. The CPTAC-Uterus dataset included 102 tumor and 15 normal samples from 101 cases. A TCGA-UCEC clinical file containing follow-up information was downloaded from the GDC.

### Gene annotation

Gene annotations were derived from GENCODE, a comprehensive resource for high-quality, evidence-based gene annotation of the human genome [23]. GENCODE v21 annotations were used for consistency, as this version was employed for gene annotation in both the TCGA-UCEC and CPTAC-Uterus datasets. GENCODE v21 classifies 19,814 genes as protein-coding genes and 7,656 as long non-coding RNAs (lncRNAs). Gene region coordinates, gene symbols, and other annotation information were obtained from the GENCODE v21 GTF file.

### Preprocessing of the datasets

Based on GENCODE v21 annotations, expression levels were extracted for all protein-coding genes and lncRNAs in both the TCGA-UCEC and CPTAC-Uterus datasets. To reduce noise, upper quartile normalization was applied to the expression data. Spearman correlation analysis revealed batch effects associated with the TCGA-UCEC and CPTAC-Uterus datasets. To mitigate these batch effects, the ComBat function from the sva R package was used [24]. ComBat employs an empirical Bayesian framework to adjust for known batch effects [16], minimizing systematic differences between datasets. In the ComBat implementation, the model matrix was specified to account for tumor versus normal sample status. Spearman correlations were visualized before and after batch effect removal to assess the effectiveness of batch correction. The resulting normalized and batch-corrected data were used for subsequent analyses.

### Identification of differentially expressed lncRNAs

To visualize the expression characteristics of protein-coding genes and lncRNAs, scatterplots were generated showing the mean expression level of each gene plotted against its squared coefficient of variation, with smoothed color density representation. To identify lncRNAs differentially expressed between tumor and normal samples in the TCGA-UCEC and CPTAC-Uterus datasets, Student’s t-tests were performed. P-values were adjusted for multiple hypothesis testing using the Benjamini-Hochberg method to control the false discovery rate (FDR). Differentially expressed lncRNAs (DE lncRNAs) were defined as those with an adjusted p-value (FDR) < 0.01 and an absolute log2 fold change ≥ 2. To increase confidence in the results, only lncRNAs exhibiting consistent up- or downregulation in both the TCGA-UCEC and CPTAC-Uterus datasets were selected for further analysis.

### Identification of survival-related lncRNAs

Due to the limited number of uncensored cases in the CPTAC-Uterus dataset (5 out of 101), which would compromise the statistical power of survival analyses, the TCGA-UCEC dataset was used to identify lncRNAs associated with patient survival. Using the differentially expressed lncRNAs (DE lncRNAs) identified previously, Cox proportional hazards (COXPH) regression models were fit to determine the association between lncRNA expression levels and patient survival. P-values from the Cox models were adjusted for multiple hypothesis testing using the Benjamini-Hochberg method to control the false discovery rate (FDR). LncRNAs with an FDR < 0.01 were considered significantly associated with survival. For visualization, Kaplan-Meier survival curves were generated by stratifying TCGA-UCEC samples into two groups based on the median expression level of each lncRNA.

### Prediction of lncRNAs’ biological functions

To infer the potential biological functions of the survival-related lncRNAs, AC025811.3 and AC012354.6 (selected based on their low FDRs in the Cox regression models), we employed a gene set enrichment approach. Given the lack of direct functional annotation for these lncRNAs, we hypothesized that their biological roles could be inferred from the protein-coding genes whose expression patterns were significantly correlated with their own. To identify these correlated genes, Spearman correlation coefficients were calculated between the expression levels of AC025811.3 and AC012354.6 and the expression levels of all protein-coding genes in the TCGA-UCEC dataset. By using the correlation matrix, the KEGG pathways [25] enriched were predicted by preranked Gene Set Enrichment Analysis (GSEA) [26]. This analysis was conducted using the fgsea package in R [27].

### Stage-stratified expression analysis

To investigate the relationship between AC025811.3 and AC012354.6 expression and disease progression, we performed stage-stratified expression analysis using the TCGA-UCEC dataset. Patients were classified into four groups based on FIGO pathological stage (Stage I, II, III, and IV). Expression levels of AC025811.3 and AC012354.6 were extracted as log2-transformed FPKM values. Differences in expression across stages were assessed using the Kruskal-Wallis test, a non-parametric method appropriate for non-normally distributed expression data. Post-hoc pairwise comparisons were performed using Dunn’s test with Benjamini-Hochberg correction for multiple testing. Additionally, Spearman correlation analysis was conducted to evaluate the association between lncRNA expression and FIGO stage treated as a continuous ordinal variable (Stage I = 1, II = 2, III = 3, IV = 4). This analysis aimed to determine whether expression levels of the survival-related lncRNAs correlate with tumor progression and could potentially serve as staging biomarkers.

## References

[1] Wright JD, Prest MT, Ferris JS, Chen L, Xu X, Rouse KJ, et al. Projected Trends in the Incidence and Mortality of Uterine Cancer in the United States. Cancer Epidemiol Biomarkers Prev. 2025;34(7):1156–66. doi: 10.1158/1055-9965.EPI-24-1422 40589281 PMC12221196

[2] Clarke MA, Devesa SS, Hammer A, Wentzensen N. Racial and Ethnic Differences in Hysterectomy-Corrected Uterine Corpus Cancer Mortality by Stage and Histologic Subtype. JAMA Oncol. 2022;8(6):895–903. doi: 10.1001/jamaoncol.2022.0009 35511145 PMC9073658

[3] Uwins C, Hablase R, Assalaarachchi H, Tailor A, Stewart A, Chatterjee J, et al. Enhanced Recovery after Uterine Corpus Cancer Surgery: A 10 Year Retrospective Cohort Study of Robotic Surgery in an NHS Cancer Centre. Cancers (Basel). 2022;14(21):5463. doi: 10.3390/cancers14215463 36358881 PMC9657636

[4] Hwang WY, Kim J-H, Noh JJ, Baek M-H, Choi MC, Lee YJ, et al. Clinical practice guidelines for uterine corpus cancer: an update to the Korean Society of Gynecologic Oncology guidelines. J Gynecol Oncol. 2025;36(1):e71. doi: 10.3802/jgo.2025.36.e71 39900346 PMC11790997

[5] Siegel RL, Miller KD, Jemal A. Cancer statistics, 2019. CA Cancer J Clin. 2019;69(1):7–34. doi: 10.3322/caac.21551 30620402

[6] Felix AS, Brinton LA. Cancer Progress and Priorities: Uterine Cancer. Cancer Epidemiol Biomarkers Prev. 2018;27(9):985–94. doi: 10.1158/1055-9965.EPI-18-0264 30181320 PMC6504985

[7] Mullins MA, Cote ML. Beyond Obesity: The Rising Incidence and Mortality Rates of Uterine Corpus Cancer. J Clin Oncol. 2019;37(22):1851–3. doi: 10.1200/JCO.19.01240 31232669 PMC6675594

[8] Wartko P, Sherman ME, Yang HP, Felix AS, Brinton LA, Trabert B. Recent changes in endometrial cancer trends among menopausal-age U.S. women. Cancer Epidemiol. 2013;37(4):374–7. doi: 10.1016/j.canep.2013.03.008 23591011 PMC3679300

[9] Masuyama H, Haraga J, Nishida T, Ogawa C, Kusumoto T, Nakamura K, et al. Three histologically distinct cancers of the uterine corpus: A case report and review of the literature. Mol Clin Oncol. 2016;4(4):563–6. doi: 10.3892/mco.2016.770 27073663 PMC4812600

[10] Tanaka T, Terai Y, Ono YJ, Fujiwara S, Tanaka Y, Sasaki H, et al. Genitofemoral neuropathy after pelvic lymphadenectomy in patients with uterine corpus cancer. Int J Gynecol Cancer. 2015;25(3):533–6. doi: 10.1097/IGC.0000000000000335 25486104

[11] Koskas M, Amant F, Mirza MR, Creutzberg CL. Cancer of the corpus uteri: 2021 update. Int J Gynaecol Obstet. 2021;155 Suppl 1(Suppl 1):45–60. doi: 10.1002/ijgo.13866 34669196 PMC9297903

[12] Rinn JL, Chang HY. Genome regulation by long noncoding RNAs. Annu Rev Biochem. 2012;81:145–66. doi: 10.1146/annurev-biochem-051410-092902 22663078 PMC3858397

[13] Gupta RA, Shah N, Wang KC, Kim J, Horlings HM, Wong DJ, et al. Long non-coding RNA HOTAIR reprograms chromatin state to promote cancer metastasis. Nature. 2010;464(7291):1071–6. doi: 10.1038/nature08975 20393566 PMC3049919

[14] Lin A, Li C, Xing Z, Hu Q, Liang K, Han L, et al. The LINK-A lncRNA activates normoxic HIF1α signalling in triple-negative breast cancer. Nat Cell Biol. 2016;18(2):213–24. doi: 10.1038/ncb3295 26751287 PMC4791069

[15] Yang M, Zhai X, Xia B, Wang Y, Lou G. Long noncoding RNA CCHE1 promotes cervical cancer cell proliferation via upregulating PCNA. Tumour Biol. 2015;36(10):7615–22. doi: 10.1007/s13277-015-3465-4 25921283

[16] Johnson WE, Li C, Rabinovic A. Adjusting batch effects in microarray expression data using empirical Bayes methods. Biostatistics. 2007;8(1):118–27. doi: 10.1093/biostatistics/kxj037 16632515

[17] Spracklen CN, Shi J, Vadlamudi S, Wu Y, Zou M, Raulerson CK, et al. Identification and functional analysis of glycemic trait loci in the China Health and Nutrition Survey. PLoS Genet. 2018;14(4):e1007275. doi: 10.1371/journal.pgen.1007275 29621232 PMC5886383

[18] Melton DA, Hrvatin S. Markers for mature beta-cells and methods of using the same. Google Patents; 2014.

[19] Varshney A, Scott LJ, Welch RP, Erdos MR, Chines PS, Narisu N, et al. Genetic regulatory signatures underlying islet gene expression and type 2 diabetes. Proc Natl Acad Sci U S A. 2017;114(9):2301–6. doi: 10.1073/pnas.1621192114 28193859 PMC5338551

[20] Chen G, Wang Z, Wang D, Qiu C, Liu M, Chen X, et al. LncRNADisease: a database for long-non-coding RNA-associated diseases. Nucleic Acids Res. 2013;41(Database issue):D983-6. doi: 10.1093/nar/gks1099 23175614 PMC3531173

[21] Ning S, Zhang J, Wang P, Zhi H, Wang J, Liu Y, et al. Lnc2Cancer: a manually curated database of experimentally supported lncRNAs associated with various human cancers. Nucleic Acids Res. 2016;44(D1):D980-5. doi: 10.1093/nar/gkv1094 26481356 PMC4702799

[22] Paraskevopoulou MD, Vlachos IS, Karagkouni D, Georgakilas G, Kanellos I, Vergoulis T, et al. DIANA-LncBase v2: indexing microRNA targets on non-coding transcripts. Nucleic Acids Res. 2016;44(D1):D231-8. doi: 10.1093/nar/gkv1270 26612864 PMC4702897

[23] Harrow J, Frankish A, Gonzalez JM, Tapanari E, Diekhans M, Kokocinski F, et al. GENCODE: the reference human genome annotation for The ENCODE Project. Genome Res. 2012;22(9):1760–74. doi: 10.1101/gr.135350.111 22955987 PMC3431492

[24] Leek JT, Johnson WE, Parker HS, Jaffe AE, Storey JD. The sva package for removing batch effects and other unwanted variation in high-throughput experiments. Bioinformatics. 2012;28(6):882–3. doi: 10.1093/bioinformatics/bts034 22257669 PMC3307112

[25] Kanehisa M, Furumichi M, Tanabe M, Sato Y, Morishima K. KEGG: new perspectives on genomes, pathways, diseases and drugs. Nucleic Acids Res. 2017;45(D1):D353–61. doi: 10.1093/nar/gkw1092 27899662 PMC5210567

[26] Subramanian A, Tamayo P, Mootha VK, Mukherjee S, Ebert BL, Gillette MA, et al. Gene set enrichment analysis: a knowledge-based approach for interpreting genome-wide expression profiles. Proc Natl Acad Sci U S A. 2005;102(43):15545–50. doi: 10.1073/pnas.0506580102 16199517 PMC1239896

[27] Sergushichev AA. 2016. 10.1101/060012

